# Integrating Artificial Intelligence into Personalized Preventive Medicine: Addressing Social Isolation and Elderly Care

**DOI:** 10.31662/jmaj.2025-0125

**Published:** 2025-11-21

**Authors:** Hakan Gocer, Ahmet Baris Durukan, Taylan Gun

**Affiliations:** 1 Department of Cardiology, Private Kutahya Park Hospital, Kütahya Park Hayat Hospital, Balikesir, Turkey; 2 Department of Cardiovascular Surgery, Ankara Liv Hospital, Ankara, Turkey; 3 Department of Cardiovascular Surgery, Istinye University School of Medicine, Istanbul, Turkey; 4 Department of Ear, Nose, and Throat, Ankara Liv Hospital, Ankara, Turkey; 5 Department of Ear, Nose, and Throat, Yuksek Ihtisas School of Medicine, Ankara, Turkey

**Keywords:** artificial intelligence, preventive medicine, personalized healthcare, machine learning, wearable technology, digital health, AI ethics, health monitoring

## Abstract

Advancements in artificial intelligence and sensor-based systems are transforming personalized preventive medicine. This “suggestion review” explores an artificial intelligence (AI)-driven healthcare ecosystem that integrates and analyzes personal health data to ensure transparency and tailored guidance for optimal well-being. AI enables real-time health monitoring, proactive interventions, and emergency response systems, addressing current healthcare limitations. By employing advanced machine learning techniques, AI improves decision-making, stress management, and personalized health recommendations. Additionally, this review explores the economic benefits of AI-driven preventive healthcare, emphasizing cost-effectiveness and improved outcomes. Ethical considerations, data security, and user autonomy are also discussed to ensure the responsible deployment of AI in healthcare.

## Introduction

The global rise in aging populations and chronic disease prevalence has exposed limitations in traditional reactive healthcare models ^(1), (2)^. Preventive medicine offers a solution, yet existing approaches often fail to deliver personalized, continuous, and accessible care. In this context, artificial intelligence (AI) has the potential to fill critical gaps by enabling predictive, real-time interventions and reducing healthcare burdens through automation. This paper presents a conceptual framework for integrating AI into preventive healthcare, particularly for elderly individuals experiencing social isolation. We define this work as a narrative review with conceptual proposals, blending narrative review methodology with forward-looking healthcare innovation.

Advancements in AI and sensor-based systems are revolutionizing personalized preventive medicine. This study explores an AI-driven healthcare ecosystem where personal health data is seamlessly integrated and analyzed, decision-making is transparent, and individuals receive tailored guidance for optimal well-being ^(1), (2)^. By assessing health data, sensor inputs, and self-reported needs, AI can regulate an individual’s environment and living conditions, issuing alerts to both the user and their physician when necessary. In emergencies, AI will automatically notify law enforcement and medical services, ensuring timely intervention ^(3), (4)^.

The need for such systems is particularly critical for the elderly population. With increasing life expectancy, the demand for home care has surged. According to the World Health Organization, the global population aged 60 years and older is expected to reach 2.1 billion by 2050, up from 1 billion in 2020 ^(3)^. This demographic shift has led to a growing preference for independent living, with many elderly individuals choosing to live alone. However, this independence often presents challenges, including difficulties in managing medications and accessing timely medical assistance.

Studies indicate that medication non-adherence among the elderly is a significant issue, with approximately 50% of older adults failing to take their medications as prescribed ^(4)^. This problem is exacerbated by cognitive decline, physical limitations, and polypharmacy (the use of multiple medications). As a result, inadequate medical support and medication errors contribute to preventable deaths. For instance, in the United States, medication-related problems among the elderly account for over 125,000 deaths annually ^(3), (4)^.

In this context, AI-driven healthcare systems offer a promising solution. By providing continuous monitoring, automated medication reminders, and real-time emergency alerts, AI can address the unique needs of the elderly population, reducing the risks associated with medication non-adherence and delayed medical interventions. This study highlights the potential of AI to transform elderly care, ensuring safer and more independent living while addressing the growing challenges of an aging population.

Advancements in artificial intelligence and sensor-based systems are also revolutionizing personalized preventive medicine ^(5)^. This study explores an AI-driven healthcare ecosystem where personal health data is seamlessly integrated and analyzed, decision-making is transparent, and individuals receive tailored guidance for optimal well-being ^(5), (6), (7)^. By assessing health data, sensor inputs, and self-reported needs, AI can regulate an individual’s environment and living conditions, issuing alerts to both the user and their doctor when necessary. In emergencies, AI will automatically notify law enforcement and medical services, ensuring timely intervention ^(7), (8), (9)^.

Several commercial systems already provide personalized health tracking, such as Apple Health, Fitbit, WHOOP, and AI-powered triage tools like Babylon Health. These platforms integrate wearable sensors and mobile applications to track physical activity, sleep, heart rate, and more. While they contribute significantly to health awareness, their current capabilities remain primarily descriptive and reactive, lacking predictive depth and integration with broader public health strategies ^(10), (11)^.

Recent advances in AI―particularly in machine learning, deep learning, and reinforcement learning―offer a promising foundation for more adaptive and anticipatory healthcare models. Deep learning algorithms can detect complex patterns in large datasets, while reinforcement learning allows systems to optimize personalized care pathways through continuous feedback and interaction. These models can be tailored to support elderly individuals by recognizing subtle deviations in physiological or behavioral data, which may indicate early signs of deterioration or social withdrawal ^(10), (11)^.

Quantum machine learning is another advancement. It provides a novel approach to deriving sophisticated results. Life-threatening diseases and toxicity require prompt, precise, and immediate detection. Rapid, accurate, and more efficient strategies that are cost-effective and non-invasive are needed, and quantum machine learning can provide such solutions ^(12)^.

Moreover, AI has been investigated in the field of drug delivery for predicting drugs embedded in formulations or layers, improving permeation, and identifying the most stable dosage forms. The conventional trial-and-error strategy in formulation development necessitates a large number of resource-intensive and time-consuming in vitro and in vivo trials. The exploration of unsupervised and supervised machine learning approaches in drug delivery may result in significant reductions in resource expenditures and significant savings in effort and time usually required for wet-laboratory trial-and-error experiments. The development stage of an AI system for drug delivery includes processing information in the input layer (gathering data), the hidden layer (training and developing a model), and the output layer (validating the model) based on specific data from repeated laboratory work. Artificial neuron networks can be employed for such purposes, and the performances of various dose forms (solubility, serum levels, hardness, etc.) and drug release can be predicted using an artificial neuron network model ^(13)^.

Similarly, AI-powered algorithms are revolutionizing cardiac failure (CF) diagnosis by utilizing large genetic, clinical, and imaging databases. To identify CF mutations quickly and precisely, machine learning methods evaluate genomic profiles. Furthermore, AI-driven imaging analysis helps to detect lung and gastrointestinal issues linked to cystic fibrosis early and allows for prompt treatment. Additionally, AI aids in individualized CF therapy by predicting how patients will respond to already available medications and enabling customized treatment regimens. Drug repurposing algorithms identify potential candidates from already approved drugs, advancing treatment options. AI also supports the optimization of pharmacological combinations, enhancing therapeutic results while minimizing side effects. it further helps with patient stratification by connecting individuals with CF mutations to therapies most suitable for their genetic profiles. This tailored strategy promises improved treatment effectiveness ^(14)^.

AI chatbots (such as those used in Babylon (www.babylonhealth.com) and Ada (https://ada.com)) are used by patients to define symptoms and receive recommendations for further actions in community and primary care settings. These can be integrated with wearable devices such as smartwatches to provide insights to both patients and caregivers for improving their behavior, sleep, and general wellness.

The emergence of ambient sensing without the need for peripherals is also noteworthy. Emerald (www.emeraldinno.com) is a wireless, touchless sensor and machine learning platform for remote monitoring of sleep, breathing, and behavior, founded by Massachusetts Institute of Technology faculty and researchers.

Google Nest claims to monitor sleep (including disturbances such as cough) using motion and sound sensors. The smart speakers can also contactlessly monitor heart rhythms.

AI systems leveraging natural language processing technology have the potential to automate administrative tasks such as documenting patient visits in electronic health records, optimizing clinical workflow, and enabling clinicians to spend more time caring for patients (e.g., Nuance Dragon Ambient eXperience (www.nuance.com/healthcare/ambient-clinical-intelligence.html)).

AI has been used to predict and prevent dengue virus infection, monitor the disease and its forecast, and support vaccine and therapeutic development as well as public health engagement ^(15)^.

Despite the dominance of deep learning in domains such as image and speech recognition, tabular data―structured data organized into rows and columns―remains central to real-world applications across healthcare, finance, and logistics. Traditional machine learning methods such as gradient boosting decision trees (GBDTs), particularly XGBoost, LightGBM, and CatBoost, have shown superior performance on tabular datasets due to their ability to model feature interactions and handle missing data robustly ^(16), (17)^. These methods are well-suited for low-dimensional, heterogeneous features that characterize tabular datasets, in contrast to the high-dimensional spatial patterns exploited by neural networks. Benchmarks consistently indicate that GBDT models outperform deep learning architectures on most tabular datasets unless extensive feature engineering is applied ^(18)^.

In response to these challenges, recent efforts have focused on adapting neural architectures for tabular data. Approaches such as TabNet and FT-Transformer attempt to incorporate inductive biases suitable for tabular structures by using attention mechanisms and feature-wise embeddings ^(19), (20)^. While these methods have shown promise, their advantages often appear in large-scale datasets or in scenarios where interpretability or scalability is critical. Moreover, hybrid models combining GBDT with deep learning elements have been proposed to leverage the strengths of both paradigms ^(20)^. As the field progresses, developing architectures that generalize across data types without sacrificing performance remains a key challenge in applying AI to tabular domains.

By integrating AI models with personalized care frameworks, more intelligent, predictive systems can be developed that not only monitor the elderly but also actively intervene before health issues escalate. This paper proposes a forward-looking conceptual framework that leverages AI to deliver targeted, preventive, and socially aware healthcare services for aging populations. While the approach is visionary, it is grounded in current technological trends and the urgent need to reimagine elderly care through proactive, data-driven solutions.

## Current Landscape

Contemporary preventive healthcare is typically limited to periodic check-ups, vaccinations, and generalized health guidelines. While effective to a degree, these strategies lack real-time adaptability and personalization. Common limitations include:

*Reactive Rather Than Proactive*: Most systems respond to illness rather than predicting and preventing it ^(6)^.

*Lack of Personalization*: Generic interventions fail to address individual metabolic, behavioral, and lifestyle differences ^(5), (6), (7)^.

*Inconsistent Health Monitoring:* The absence of continuous, real-time tracking creates gaps in disease prevention ^(8), (21)^.

*Limited Emergency Integration:* Few systems are equipped to detect emergencies autonomously and alert appropriate responders ^(21)^.

*Neglect of Social Determinants:* Isolation, loneliness, and environmental stressors are rarely integrated into care plans ^(4), (5)^.

The proposed AI-driven model addresses these issues through real-time health monitoring, predictive analytics, automated alerts, and personalized lifestyle guidance―particularly for at-risk elderly individuals.

## Proposed AI Ecosystem

### AI and personalized preventive medicine

AI technologies―particularly deep learning, reinforcement learning, and Bayesian inference―allow for continuous, adaptive analysis of multimodal health data. Key functionalities include:

Pattern Recognition & Risk Prediction: Algorithms analyze biometric, behavioral, and environmental data to identify subtle health trends ^(7), (9), (22), (23)^.

Explainable AI: Decision trees, interpretable neural networks, and transparent Bayesian models enhance clinical trust and user understanding ^(23), (24)^.

Contactless Monitoring: Thermal and RGB cameras detect early signs of illness, such as fever or respiratory anomalies ^(7), (8), (9), (25), (26)^.

In-Home Diagnostics: Smart toilets and AI-powered biosensors assess urine/stool samples to optimize nutrition and detect early disease markers ^(7), (8), (9), (21), (27)^.

Additionally, reinforcement learning allows AI systems to continuously refine decisions based on new inputs, user feedback, and health outcomes. Federated learning ensures that personalized models can be trained across distributed data sources (e.g., multiple households or clinics) while maintaining user privacy.

### Digital health, social isolation, and stress management

AI systems can detect and mitigate stress and social isolation through various means:

Voice and Facial Expression Analysis: Detects emotional distress and offers coping strategies ^(12), (14), (28), (29), (30)^.

Behavioral Pattern Recognition: Identifies signs of depression, loneliness, or cognitive decline ^(14), (28), (29), (30)^.

Virtual Companionship and Reminders: AI-driven digital assistants provide interactive engagement, medication prompts, and emotional support ^(11), (16), (31)^.

Environment Regulation: AI adjusts lighting, temperature, and noise to improve psychological comfort and support circadian rhythms ^(21), (23), (29)^.

By incorporating psychosocial variables, AI systems extend beyond physical health to support emotional and mental well-being.

### Integrated AI infrastructure and personalized health management

A comprehensive AI-based ecosystem requires a multilayered infrastructure, integrating diverse components:

Sensor-Based Data Collection: Wearables and home devices collect real-time health data (heart rate, SpO2, activity levels) ^(31)^.

Modular System Design: Subsystems (e.g., smart fridges, beds, toilets) communicate through a centralized AI brain ^(10)^.

Personalized Nutrition and Lifestyle Guidance: AI tailors diets and routines to match metabolic needs, preferences, and health status ^(28)^.

Adaptive Learning: Feedback-driven machine learning enables continuous system improvement over time ^(10), (31)^.

### Emergency response and autonomous support

AI systems respond swiftly to anomalies, including:

Real-Time Alerting: Health anomalies trigger alerts to users, caregivers, or healthcare providers ^(31)^.

Autonomous Emergency Activation: AI contacts emergency services automatically when critical thresholds are breached ^(11), (31)^.

Fall Detection and Response: Smart cameras and floor sensors identify falls or immobility, initiating immediate intervention.

These features are crucial for elderly users, particularly those living alone or with mobility issues.

### Cost-effectiveness in preventive healthcare

While initial setup costs for AI-driven systems are substantial, long-term economic benefits include:

Early Detection: Reduces severity and cost of treatment through timely intervention ^(10)^.

Lower Hospitalization Rates: Continuous monitoring helps prevent complications and avoid unnecessary admissions ^(10)^.

Optimized Resource Allocation: Streamlined decision-making and automation reduce clinician burden ^(31)^.

Efficient Medication Management: Automated adherence systems reduce prescription errors and wastage ^(11)^.

Macroeconomic Gains: Healthier populations yield higher productivity and reduced long-term care expenses ^(27)^.

### Ethical considerations

The responsible deployment of AI in healthcare must address ethical and experiential concerns:

Data Privacy: Federated learning minimizes centralized data risks, enhancing user trust.

Algorithmic Bias: Transparent validation across diverse datasets prevents discriminatory outcomes.

User Autonomy: Individuals can calibrate AI assistance levels, ensuring they retain control over health decisions ^(31)^.

Stress-Sensitive Design: Intervention frequency is adjusted based on user stress levels, avoiding cognitive overload ^(11)^.

Ethical foresight is essential for gaining user acceptance and regulatory approval.

## Discussion

This study presents a conceptual framework for integrating AI into personalized preventive healthcare, with a particular emphasis on elderly individuals and socially isolated populations. The proposed AI-driven model leverages advanced AI techniques such as reinforcement learning, federated learning, and explainable algorithms to enable real-time health monitoring, adaptive interventions, and early detection of both physical and psychosocial risks ^(30), (31)^. By addressing not only physiological parameters but also emotional well-being and social interaction, this approach supports a more holistic understanding of health.

Particularly in aging societies, where loneliness and chronic disease frequently co-exist, AI systems that can detect behavioral changes and trigger timely support could significantly reduce disease burden and improve the quality of life for elderly individuals ^(3), (5), (6)^. The integration of AI in healthcare provides a transformative opportunity to shift from reactive treatment to proactive and personalized prevention ^(11), (21), (31)^. AI-driven systems can help reduce healthcare costs, enhance autonomy, and mitigate risks by enabling early intervention and personalized care

However, challenges remain that must be addressed for the successful implementation of these systems. Key concerns include ensuring algorithmic transparency, addressing data privacy issues, and making AI solutions accessible to non-digital populations ^(10), (21), (29), (30)^. Additionally, regulatory frameworks are required to ensure safety, fairness, and equity in the deployment of AI in healthcare. These obstacles should be considered in ongoing interdisciplinary research spanning medicine, data science, ethics, and engineering, to refine algorithms and improve sensor technology ^(11), (21)^.

The integration of AI into personalized preventive healthcare, particularly for elderly populations, presents immense potential but also several implementation challenges. While existing commercial systems such as Apple Health, Fitbit, and Babylon Health have laid foundational work in consumer-level health monitoring, they often fall short in delivering truly adaptive and proactive interventions. Most of these platforms operate on retrospective data and offer reactive insights rather than predictive and context-aware decision-making ^(11), (21), (28), (31)^.

One critical limitation in current AI-driven real-time decision-making systems is their dependency on static or narrowly contextualized datasets, which limits their responsiveness to dynamic changes in an individual’s health or social environment. These systems often lack the ability to integrate multisource data―such as physiological parameters, behavioral patterns, and environmental cues―in a cohesive and temporally sensitive manner. Moreover, the decision algorithms deployed are frequently optimized for generalized populations rather than personalized, evolving health profiles ^(11), (21), (28), (31)^.

Another gap lies in the limited interpretability and transparency of AI models in clinical settings. For elderly care, where decisions may involve ethical, cognitive, and psychosocial dimensions, opaque AI decisions may hinder adoption by caregivers or healthcare providers. Additionally, real-time applications demand low-latency processing and high accuracy, which remains technically challenging in decentralized or resource-constrained environments such as home care settings ^(11), (21), (31)^.

The model proposed in this paper addresses these limitations by advocating for an AI ecosystem that integrates reinforcement learning and deep learning algorithms with continuous feedback loops. This framework would allow systems to learn from individual user data over time, adapting interventions not only to medical conditions but also to social behaviors and psychological cues. Moreover, by combining data from wearables, smart home devices, and digital interactions, the proposed model emphasizes real-time, holistic monitoring that is both predictive and socially sensitive ^(10), (21), (30)^.

Importantly, the model incorporates a layer of ethical oversight and explainability, enabling stakeholders―patients, caregivers, and clinicians―to understand the rationale behind AI-driven recommendations. This is especially crucial in promoting trust and adoption among older adults, many of whom may have limited digital literacy or skepticism toward automated systems ^(10), (11), (31)^. As healthcare systems worldwide transition toward value-based care models, the proactive and personalized interventions facilitated by such an AI framework can significantly reduce hospitalization rates, improve quality of life, and alleviate the economic burden of aging populations ^(11), (21), (31)^.

The potential of AI in transforming healthcare is immense, particularly in elder care, where it can offer continuous monitoring, adaptive learning, and real-time alerts. However, its widespread adoption will require careful consideration of ethical, regulatory, and technical challenges.

## Conclusions

This study proposes a scalable and modular AI-driven preventive healthcare model designed to support individualized interventions, prioritize user well-being, and cater specifically to aging populations. The integration of reinforcement learning, federated learning, and real-time monitoring ensures that the system is adaptive, privacy-preserving, and capable of making predictive decisions that can enhance the health outcomes of users.

Future healthcare systems should evolve into holistic care ecosystems where AI not only supports personal health but also contributes to broader public health objectives. This can include early outbreak detection, population health analytics, and resource optimization. To realize this vision, ongoing interdisciplinary collaboration is essential, particularly in the areas of algorithm development, sensor technology, and ensuring that AI-powered solutions are accessible to vulnerable and underserved populations.

If developed responsibly and ethically, AI has the potential to revolutionize healthcare, transitioning the focus from reactive treatment to proactive, personalized prevention. This would not only pave the way for longer and healthier lives but also improve the autonomy of individuals, particularly the elderly, enhancing their quality of life through technology-driven, personalized care.

## Article Information

### Author Contributions

The conception of the work, or the acquisition, analysis, or interpretation of data for the work: Hakan Gocer, Ahmet Baris Durukan, Taylan Gun.

Drafting the work, reviewing it critically for important intellectual content: Hakan Gocer, Ahmet Baris Durukan, Taylan Gun.

Final approval of the version to be published: Hakan Gocer, Ahmet Baris Durukan, Taylan Gun.

Agreement to be accountable for all aspects of the work in ensuring that questions related to the accuracy or integrity of any part of the work are appropriately investigated and resolved: Hakan Gocer, Ahmet Baris Durukan, Taylan Gun.

### Conflicts of Interest

None

### IRB Approval Code and Name of the Institution

Not applicable.
